# “WE’RE CAMPAIGN OBJECTS”: A QUALITATIVE LIVELIHOOD ANALYSIS OF THE VISUALLY IMPAIRED OLDER ADULT IN RURAL GHANA

**DOI:** 10.1093/geroni/igae098.2681

**Published:** 2024-12-31

**Authors:** Eric Frimpong, Akwasi Adjei Gyimah, Mathias Adjei

**Affiliations:** University of Massachusetts Boston, Boston, Massachusetts, United States; Miami University, Oxford, Ohio, United States; Miami University, Oxford, Ohio, United States

## Abstract

**Background:**

In Ghana, a considerable proportion of the older adult population continue to face economic hardship due to the weakening of family structures and inadequate social support. This study aims to investigate the lived experiences of visually impaired older individuals residing in rural areas of the country.

**Methods:**

For a deeper understanding of the experiences of respondents, the social model of disability informed the use of a qualitative approach to interviewing a total of 18 older adults residing in selected rural areas in Ghana. The data was analyzed through a thematic content analysis approach. Findings: Many older adults in Ghana living with visual impairment continued to experience economic hardship. They often rely on donations from family members and social groups, such as churches. However, these sources of support are inadequate to meet their needs. A major concern raised was access to government support programs. For those who were enrolled in such programs, they expressed dissatisfaction with the benefits received, citing their insufficiency in meeting their needs. Furthermore, many respondents expressed disillusionment with politicians who were perceived to have made empty promises during election campaigns in an attempt to implement programs that will enhance their livelihood.

**Conclusion:**

These findings illustrate the pressing need for more effective support systems and interventions to address the economic challenges faced by visually impaired older people in rural Ghana. Policymakers must consider the unique needs and experiences of this population, and work towards implementing solutions that are both sustainable and effective.

